# Association of Frailty with Adverse Outcomes in Patients with Suspected COVID-19 Infection

**DOI:** 10.3390/jcm10112472

**Published:** 2021-06-02

**Authors:** Noemi R. Simon, Andrea S. Jauslin, Marco Rueegg, Raphael Twerenbold, Maurin Lampart, Stefan Osswald, Stefano Bassetti, Sarah Tschudin-Sutter, Martin Siegemund, Christian H. Nickel, Roland Bingisser

**Affiliations:** 1 Emergency Department, University Hospital Basel, 4031 Basel, Switzerland; noemi.simon@usb.ch (N.R.S.); andrea.jauslin@usb.ch (A.S.J.); marco.rueegg@usb.ch (M.R.); christian.nickel@usb.ch (C.H.N.); 2 Department of Cardiology, University Hospital Basel, 4031 Basel, Switzerland; raphael.twerenbold@usb.ch (R.T.); maurin.lampart@usb.ch (M.L.); stefan.osswald@usb.ch (S.O.); 3 Department of Internal Medicine, University Hospital Basel, 4031 Basel, Switzerland; stefano.bassetti@usb.ch; 4 Division of Infectious Disease & Hospital Epidemiology, University Hospital Basel, 4031 Basel, Switzerland; sarah.tschudin@usb.ch; 5 Department of Clinical Research, University of Basel, C/O University Hospital Basel, 4031 Basel, Switzerland; martin.siegemund@usb.ch; 6 Department of Intensive Care, University Hospital Basel, 4031 Basel, Switzerland

**Keywords:** age, frailty, COVID-19, SARS-CoV-2, mortality, intensive care, emergency department

## Abstract

Older age and frailty are predictors of adverse outcomes in patients with COVID-19. In emergency medicine, patients do not present with the diagnosis, but with suspicion of COVID-19. The aim of this study was to assess the association of frailty and age with death or admission to intensive care in patients with *suspected* COVID-19. This single-centre prospective cohort study was performed in the Emergency Department of a tertiary care hospital. Patients, 65 years and older, with *suspected* COVID-19 presenting to the Emergency Department during the first wave of the pandemic were consecutively enrolled. All patients underwent nasopharyngeal SARS-CoV-2 PCR swab tests. Patients with a Clinical Frailty Scale (CFS) > 4, were considered to be frail. Associations between age, gender, frailty, and COVID-19 status with the composite adverse outcome of 30-day-intensive-care-admission and/or 30-day-mortality were tested. In the 372 patients analysed, the median age was 77 years, 154 (41.4%) were women, 44 (11.8%) were COVID-19-positive, and 125 (33.6%) were frail. The worst outcome was seen in frail COVID-19-patients with six (66.7%) adverse outcomes. Frailty (CFS > 4) and COVID-19-positivity were associated with an adverse outcome after adjustment for age and gender (frailty: OR 5.01, CI 2.56–10.17, *p* < 0.001; COVID-19: OR 3.47, CI 1.48–7.89, *p* = 0.003). Frailty was strongly associated with adverse outcomes and outperformed age as a predictor in emergency patients with *suspected* COVID-19.

## 1. Introduction

Older age is associated with adverse outcomes in COVID-19 patients [1,2,3]. Frailty, on the other hand, appears to be a predictor for adverse outcomes in hospitalised COVID-19 patients [4,5,6,7]. The Clinical Frailty Scale (CFS) is a validated tool to assess frailty, and has already been implemented in emergency settings [8,9,10,11,12]. There is a debate if frailty could be of higher prognostic importance than age [13,14,15,16,17,18]. Previous meta-analyses addressing the importance of frailty in hospitalised COVID-19 patients showed conflicting results [19,20].

To our knowledge, no prospective emergency cohorts with *suspected* COVID-19, comparing COVID-19 patients with controls, have been published. This is important to emergency department (ED) management, as COVID-19 is often only a suspicion or a working hypothesis. Early decisions regarding triage and resource allocation have to be taken before test results are available [21]. While the COVID-19 status of ED patients with fever and respiratory symptoms is often unknown, age and frailty status can be determined at presentation [8].

Therefore, we intended to assess the association between age, frailty, and adverse outcomes in patients presenting with *suspected* COVID-19 during the first wave of the pandemic. Second, we planned to compare COVID-19 *positive* with COVID-19 *negative* patients, hypothesizing that frailty is more important than age for the prognosis of patients with *suspected* COVID-19.

## 2. Materials and Methods

### 2.1. Study Design, Population and Inclusion Criteria

This study is part of a prospective, observational, cohort COronaVIrus surviVAl (COVIVA, ClinicalTrials.gov NCT04366765) study including unselected ED visits of patients aged 18 years and older presenting with suspected severe acute respiratory syndrome coronavirus 2 (SARS-CoV-2) infection to the emergency department of the University Hospital in Basel, Switzerland, during the first wave of the COVID-19 pandemic between March 2020 and June 2020.

SARS-CoV-2 infection was suspected in any patient with breathlessness, other respiratory symptoms or flu-like symptoms, such as fever, chills, sore throat, cough, myalgia, and headache [22]. In addition, patients with acute confusion or general deterioration (i.e., weakness and abnormal fatigue) were considered, if these symptoms could not be explained otherwise. Of note, during the first few weeks of the pandemic, hyposmia/anosmia or hypogeusia/ageusia was not systematically assessed as a predictor of COVID-19-positivity, as these predictors emerged later.

The University Hospital of Basel is a tertiary care centre with approximately 54,000 annual ED visits. In the beginning of the first wave of the pandemic, we established a Triage and Test Centre (TTC) in a nearby church (30-m walking distance to the ED) where all patients with suspected COVID-19 were tested for the presence of SARS-CoV-2. Patients with a National Early Warning Score (NEWS) > 2, oxygen saturation < 94%, or a higher disease severity rating by physicians were directly referred to the ED [22,23].

All patients underwent cross-validated nasopharyngeal SARS-CoV-2 polymerase chain reaction (PCR) swab tests [22,24,25]. Patients were considered COVID-19 *positive* if one or multiple SARS-CoV-2 PCR swab tests (between 14 days prior to or post ED presentation) were *positive*. All patients with *negative* SARS-CoV-2 PCR swab test results were included and observed as *controls*. All participating patients or their legally authorized representatives gave written general consent. The study was conducted according to the principles of the Declaration of Helsinki, approved by the local ethics committee (identifier EKNZ 2020-00566).

In this analysis, all patients aged 65 years and older were consecutively enrolled. In case of multiple ED presentations, only the index presentation was analysed. The authors designed the study, gathered, and analysed the data according to the STROBE guidelines [26].

### 2.2. Clinical Assessment

Symptoms were assessed by a standardised questionnaire filled in by patients at the time of ED presentation. All patients underwent a thorough clinical assessment by the ED physician in charge according to local standard operating procedures. Vital parameters, including body temperature, heart rate, blood pressure, oxygen saturation, and breathing rate, were assessed in every patient.

Patients were admitted to the Intensive Care Unit (ICU) if they were in need of respiratory support, if they were clinically unstable (e.g., in need of catecholamine therapy), or if they were of reduced vigilance and if an ICU-admission was in accordance with the patient’s preferences.

The patient management was left at the discretion of the ED physicians.

### 2.3. Adjudication of Final Diagnosis

To determine the final diagnosis that led to the index ED presentation and the clinical suspicion of COVID-19, trained physicians reviewed all medical data available including 30-days post-discharge follow-up information and chose from a predefined list of diagnoses that best fit each patient. Predefined main categories included, but were not limited to, COVID-19, non-SARS-CoV-2 infections (e.g., other respiratory, gastrointestinal, and urogenital), cardiovascular disease (acute coronary syndrome, rhythm disorder, congestive heart failure, and pulmonary embolism), other pulmonary non-infectious disease (e.g., lung tumour, asthma, and chronic obstructive pulmonary disease), and neurologic disease (e.g., stroke and seizure).

### 2.4. Clinical Frailty Scale (CFS)

The Clinical Frailty Scale is an easy-to-use tool to assess the frailty level two weeks prior to ED presentation. The CFS ranks frailty numerically from 1 to 9 (1 very fit, 2 fit, 3 managing well, 4 living with very mild frailty, 5 living with mild frailty, 6 living with moderate frailty, 7 living with severe frailty, 8 living with very severe frailty, and 9 terminally ill). Each CFS-level comes with a short description and a pictograph [27]. According to local standard operating procedure, the CFS is assessed in every patient aged 65 years and older.

For this analysis, levels of the CFS were grouped with 1–4 being “not frail” and 5–9 being “frail”. All eligible patients were assigned a frailty level according to the German version of the Clinical Frailty scale [8].

### 2.5. Outcome Measures

At 30 days after discharge, patients were contacted by telephone calls or in written form by research physicians or study nurses and information about their current health, hospitalisations, and adverse events were obtained, guided by a predefined set of questions and itemised checklists. Records of hospitals and primary care physicians, as well as death registries were reviewed for additional information.

The primary outcome of this study was the composite endpoint of all-cause mortality (death within 30 days after ED presentation), and/or admission to an ICU (ICU-admission within 30 days after ED presentation) as an adverse outcome. Secondary endpoints were admission to a medical ward, admission to an ICU, invasive mechanical ventilation, and 30-day-mortality.

### 2.6. Primary Data Analysis

Descriptive statistics are presented as counts and frequencies for categorical data, and median [interquartile range] for metric variables. Kruskal–Wallis tests were used for comparisons of medians, and chi-squared or exact Fisher tests in cells with expected frequencies below *n* = 5.

Logistic regressions were used to calculate the odds ratios (OR), 95% Confidence Intervals (CI), and *p*-values and were adjusted for age and gender. In order to compare time to event data, Cox proportional hazards regression models, adjusted for age and gender, were performed. If the proportional hazard was not met, then the Cox-regression was stratified for combined groups (CFS and COVID-19 status). For a stratified Cox-regression, it was not possible to compare between subgroup levels. However, adjustment for age and gender was feasible for each strata and was used for graphical description.

A *p*-value < 0.05 was considered significant. All evaluations were performed using the statistical software R version 4.0.3 (https://cran.r-project.org/bin/windows/base/ (accessed on 10 October 2020)).

## 3. Results

### 3.1. Baseline Characteristics

**Figure 1:**
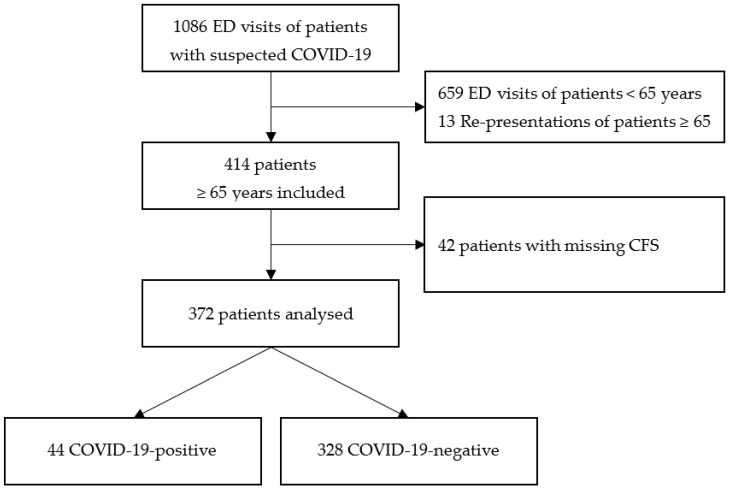
Inclusion procedure of patients with suspected COVID-19 who presented to the ED.

Of 427 ED visits of patients aged 65 years and older with *suspected* COVID-19, 55 (12.9%) were excluded due to missing data or re-presentation. Therefore, the final study population consisted of 372 patients with *suspected* COVID-19 (see Figure 1). Comparison of patients analysed and patients excluded due to missing CFS is shown in Table A1.

**Figure A1:**
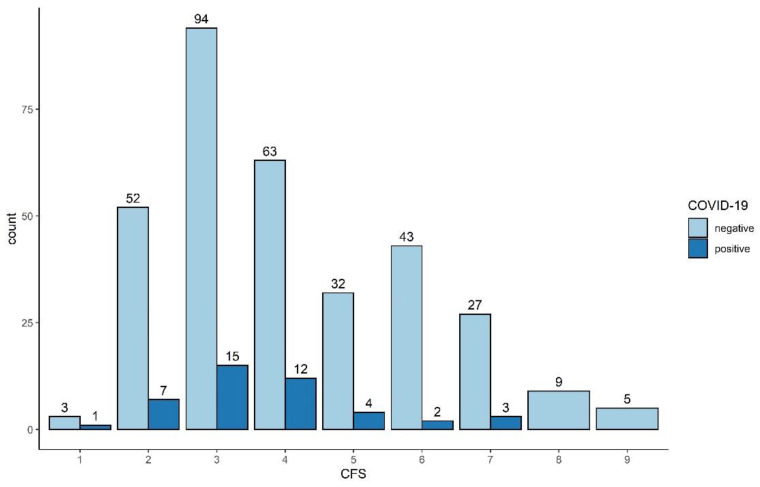
Distribution of CFS-levels in COVID-19 and non-COVID-19 patients.

269 (72.3%) patients were self-referrals, and 103 (27.7%) were Emergency Medical Services (EMS) referrals. Baseline characteristics of all patients analysed are shown in Table 1. The median age was 77 years [IQR 71; 83 years], 154 (41.4%) were women. 44 (11.8%) patients had a positive SARS-CoV-2 PCR test result, and 125 (33.6%) patients were frail (CFS > 4). The distribution of single CFS levels divided in COVID-19 status are shown in Figure A1. The final diagnoses of all patients with *suspected* COVID-19 and subsequent *negative* PCR swab test are presented in Table A2.

Of all 44 patients with positive SARS-CoV-2 PCR swab test results, 9 (20.5%) were frail, as compared to 116 (35.4%) controls. There was no significant difference in COVID-19-patiens and non-COVID-19-patients regarding vital signs at time of ED presentation, and COVID-19-patients were less likely to report dyspnoea and weakness (see Table 1).

### 3.2. Patients with Suspected COVID-19

**Figure 2:**
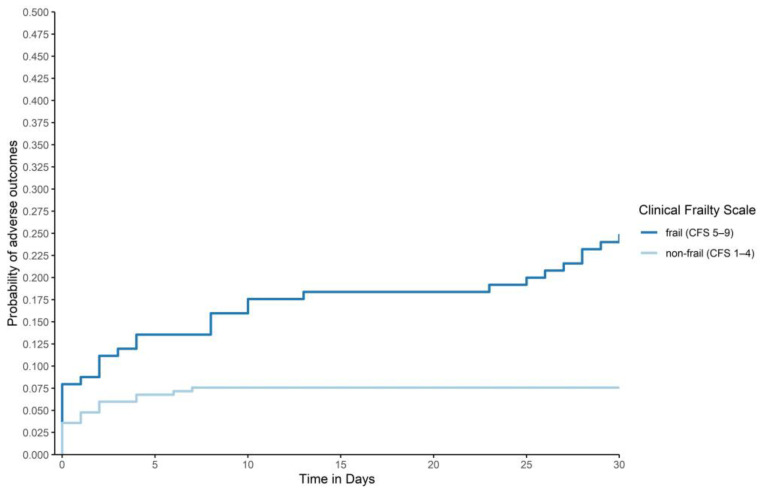
Adverse outcomes by frailty status: Graphical description of adverse outcomes (30-day-mortality or 30-day-ICU-admission) based on Cox regression with frailty status as strata, adjusted for age and gender. Frailty-levels were collapsed to “frail” (CFS 5–9) and “non-frail” (CFS1–4). To improve readability, the graph was cropped to 0.5 on the y axis. (*p* < 0.001, *p*-value based on logistic regression).

In frail patients with *suspected* COVID-19, 32 (25.6%) adverse outcomes (primary composite outcome of 30-day-mortality or 30-day-ICU-admission) were reported, as compared to 19 (7.7%) in non-frail patients (*p* < 0.001 based on logistic regression). These results are graphically presented in Figure 2.

In patients with *suspected* COVID-19, frailty and a positive SARS-CoV-2 PCR swab test were associated with an adverse outcome after adjusting for age and gender (frailty: OR 5.01, CI 2.56–10.17, *p* < 0.001; COVID-19: OR 3.47, CI 1.48–7.89, *p* = 0.003) (see Table 2).

Adjusted for age and gender, a positive SARS-CoV-2 PCR swab test was associated with a higher 30-day-mortality (OR 3.54, CI 1.14–10.16, *p* = 0.021), ICU-admission (OR 3.41, CI 1.24–8.63, *p* = 0.012), and mechanical ventilation (OR 7.10, CI 1.31–35.4, *p* = 0.015), as secondary outcomes in patients with *suspected* COVID-19 (see Table 2). Generally, frailty was associated with a higher risk of 30-day-mortality (OR 6.92, CI 2.75–19.54, *p* < 0.001), admission to ICU (OR 2.37, CI 1.00–5.67, *p* = 0.049), and admission to a medical ward (OR 3.59, CI 2.03–6.64, *p* < 0.001), after adjusting for age and gender.

Age was significantly associated with admission to a medical ward (OR 1.05, CI 1.02–1.09, *p* = 0.002). No significant association was seen between age and the composite outcome, age and 30-day-mortality, age and ICU-admission, as well as age and mechanical ventilation. Gender did not have a significant impact on either the primary or any of the secondary outcomes in patients with *suspected* COVID-19 (see Table 2).

### 3.3. Patients with Confirmed COVID-19 and Controls

**Figure 3:**
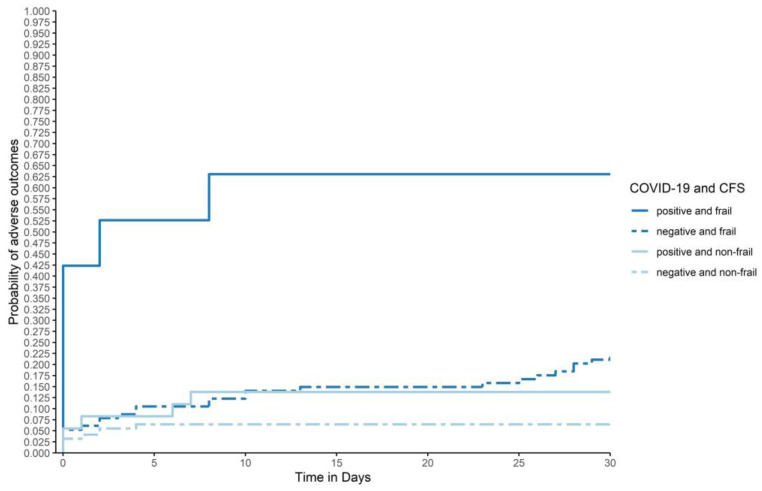
Adverse outcomes by frailty and swab status: Graphical description of adverse outcomes (composite outcome of 30-day-mortality or 30-day-ICU-admission), based on a Cox regression with combined groups of COVID-19 and CFS-status as strata, adjusted for age and gender, within the first 30 days after ED-admission for all patients in each subgroup (COVID-19-positive and frail, COVID-19-negative and frail, COVID-19-positive and non-frail, and COVID-19-negative and non-frail; *n* = 372). Frailty-levels were collapsed to “frail” (CFS 5–9) and “non-frail” (CFS1–4). (*p* < 0.001, *p*-value based on logistic regression).

The worst outcome was seen in frail, COVID-19-patients with six (66.7%) adverse outcomes, followed by frail non-COVID-19-patients (*n* = 26, 22.4%). In non-frail COVID-19- patients, five (14.3%) adverse outcomes were reported. The best outcome was seen in non-frail non-COVID-19 patients with 14 (6.6%) adverse outcomes (*p* < 0.001, based on logistic regression). These results are graphically presented in Figure 3.

In patients with *confirmed* COVID-19, 11 (25.0%) adverse outcomes were documented, as compared to 40 (12.2%) in non-COVID-19-patients (*p* = 0.037). Outcomes in COVID-19-patients as compared to non-COVID-19-patients are shown in Table 1. After adjusting for age and gender, frailty was independently associated with adverse outcomes in patients with *confirmed* COVID-19 (OR 4.17, CI 2.19–8.14, *p* < 0.001). Gender and age did not have a significant impact on adverse outcomes of COVID-19-patients (age: OR 0.99, CI 0.95–1.03, *p* = 0.614; female gender: OR 1.28, CI 0.69–2.38, *p* = 0.435).

## 4. Discussion

The main results of the study were the high odds for adverse outcomes in patients presenting with *suspected* COVID-19 and frailty, the striking similarity between COVID-19 affected and non-affected patients regarding demographics and initial disease severity, and the prognostic power of frailty, and COVID-19 status in the entire cohort.

In detail: The composite primary endpoint was highly associated with both frailty and the presence of COVID-19. Independent of age and gender, the group with the worst outcome (frail, COVID-19-positive) was well separated from the intermediate outcome group (frail, COVID-19-negative, and non-frail, COVID-19-positive), and the group with the best outcome (non-frail, COVID-19-negative). Overall mortality was associated with frailty status in age- and gender-adjusted analyses. Age was only associated with admission to a medical ward, and gender was not associated with any primary or secondary outcome.

The new information provided by this cohort-study is the direct comparison between older COVID-19 *positive* and *negative* patients with comparable presenting symptoms and disease severity in a highly standardized setting [22]. Our main interest was the predictive power of frailty status, particularly when adjusted for age, in all patients presenting with *suspected* COVID-19. The results show that, in both COVID-19 *positive* and *negative* older patients, frailty may be taken as a predictor of adverse outcomes. The composite endpoint reflecting adverse outcome seems adequate, considering the high burden of morbidity post intensive care treatment, particularly in older patients, mortality itself reflecting only the tip of the iceberg [28,29].

Most previous studies on the topic have shown data from hospitalised patients or specialized units [4,5,6,7,14,15,16,17,18,19,20,30,31,32,33,34,35]. Frailty status could be helpful for resource allocation, due to high odds for adverse outcomes. The assessment of the frailty status has been facilitated by the use of the CFS, which is a quick and easy tool [36].

The missing association of age with intensive care admission and death could be explained by a certain reluctance toward invasive therapy in this older and rather frail population. However, age by itself was not associated with death, and only hospitalisation was associated with age in this group of patients over 65. As frailty is a concept validated only for older age, the whole cohort of 1086 patients (showing age dependent mortality) could not be analysed for the effects of frailty.

The higher association of frailty with admission to a medical ward rather than with ICU-admission and mechanical ventilation might be due to patients’ preferences. Frail patients might well disagree with ICU admission and/or mechanical ventilation but agree with admission to a medical ward. Importantly, we have not used frailty for disposition decisions to intensive care, as the effects of frailty were unknown during the first wave. For new guidelines on resource allocation under critical circumstances, this finding could be important.

Taken together, the hypothesis that frailty may have a higher association with adverse outcomes than age in older ED patients *suspected* of COVID-19 is supported by our data. We, therefore, suggest using a simple tool, such as the CFS, to evaluate frailty at presentation for forecasting and organizing resources, disposition, and communication with patients and their proxies.

### Strengths and Limitations

A major strength of this study is the comparison of patients presenting with *suspected* COVID-19 and subsequent *positive* SARS-CoV-2 PCR swab tests to patients with similar symptoms but *negative* swab tests. With the Clinical Frailty Scale, we used a validated tool with good interrater reliability [8,9,10,11,12]. Since all patients aged 65 years and older with *suspected* COVID-19 infection and frailty level assessment were included, we minimised the risk of a selection bias. However, generalisability is limited due to the monocentric study design.

In addition, our sample size was limited to patient-referrals from the first wave of the pandemic. Therefore, a relatively small number of COVID-19-positive older patients was analysed, and few adverse outcomes were registered. The comparability of adverse outcomes stratified by frailty and COVID-19 status in Figure 3 is hence limited. Second, anosmia/hyposmia and ageusia/hypogeusia were not assessed in this analysis. Third, 10% of all older patients had to be excluded for missing CFS-levels. Frailty was less likely to be evaluated in patients with very high acuity, who were less likely to be included. Additionally, a comorbidity score, the patient’s preferences for ICU-admission or mechanical ventilation, and complications, such as secondary infections or thromboembolic events, were not assessed. Therefore, the interaction of these variables with the primary or one of the secondary outcomes could not be evaluated. Furthermore, the study was insufficiently powered to assess some secondary analyses, such as hospital length of stay, or rehospitalisation.

## 5. Conclusions

In emergency patients with *suspected* COVID-19-infection, frailty is associated with adverse outcomes (30-day-ICU-admission or 30-day-mortality). Frailty outperformed age as a predictor for these outcomes in all patients with *suspected* COVID-19, as well as in the subgroup of patients with *confirmed* COVID-19. Frailty should be considered as a predictor for adverse outcomes in patients with *suspected* COVID-19 at ED presentation.

## Figures and Tables

**Table 1:** Characteristics of ED patients aged 65 years and older at time of ED presentation.

	All (*n* = 372)		COVID-19 Negative (*n* = 328)		COVID-19 Positive (*n* = 44)		*p*-Value	N
Age, median (IQR)	77.0	(71.0; 83.0)	77.0	(71.0; 83.0)	77.0	(72.0; 85.0)	0.760	372
Female gender, n (%)	154	(41.4)	133	(40.5)	21	(47.7)	0.456	372
Frailty (CFS > 4), n (%)	125	(33.6)	116	(35.4)	9	(20.5)	0.072	372
Dyspnoea, n (%)	167	(44.9)	151	(46.0)	16	(36.4)	0.294	372
Confusion, n (%)	63	(16.9)	56	(17.1)	7	(15.9)	1.000	372
Weakness, n (%)	113	(30.4)	106	(32.3)	7	(15.9)	0.041	372
Abnormal Fatigue, n (%)	152	(40.9)	133	(40.5)	19	(43.2)	0.865	372
ESI level, n (%)							0.012	367
1	11	(3.0)	8	(2.5)	3	(7.0)		
2	195	(53.1)	182	(56.2)	13	(30.2)		
3	157	(42.8)	130	(40.1)	27	(62.8)		
4	4	(1.1)	4	(1.2)	0	(0.0)		
5	0	(0.0)	0	(0.0)	0	(0.0)		
Temperature, (°C), median (IQR)	37.1	(36.6; 38.1)	37.1	(36.5; 38.1)	37.2	(36.8; 38.0)	0.687	354
Respiratory rate (brpm), median (IQR)	20.0	(16.0; 24.0)	20.0	(16.0; 24.0)	20.0	(16.0; 25.0)	0.776	358
Oxygen saturation (%), median (IQR)	96.0	(94.0; 97.0)	96.0	(94.0; 97.0)	95.0	(94.0; 96.5)	0.093	361
Heart rate (bpm), median (IQR)	86.0	(74.0; 100.0)	86.5	(73.0; 101.0)	83.0	(75.5; 97.0)	0.450	361
Systolic BP (mmHg), median (IQR)	138.0	(120.0; 159.0)	140.0	(121.0; 160.0)	131.0	(119.0; 151.0)	0.119	356
Diastolic BP (mmHg), median (IQR)	78.0	(67.0; 86.0)	79.5	(68.0; 86.0)	71.0	(64.8; 81.2)	0.153	350
NEWS, median (IQR)	3.0	(1.0; 5.0)	3.0	(1.0; 5.0)	3.0	(2.0; 5.0)	0.747	354
Initial disposition, n (%)							0.175	372
Outpatient	94	(25.3)	86	(26.2)	8	(18.2)		
Medical ward	256	(68.8)	225	(68.6)	31	(70.5)		
ICU	22	(5.9)	17	(5.2)	5	(11.4)		
30-day-ICU-admission, n (%)	27	(7.3)	20	(6.1)	7	(15.9)	0.029	372
Mechanical ventilation, n (%)	7	(1.9)	4	(1.2)	3	(6.8)	0.038	372
30-day-mortality, n (%)	27	(7.3)	21	(6.4)	6	(13.6)	0.103	372
Composite outcome, n (%)	51	(13.7)	40	(12.2)	11	(25.0)	0.037	372

The table comprises all patients analysed, patients with subsequent negative SARS-CoV-2 PCR swab tests and patients with subsequent positive SARS-CoV-2 PCR swab tests. ED = emergency department; SARS-CoV-2 = severe acute respiratory syndrome coronavirus 2; PCR = polymerase chain reaction; IQR = interquartile range (25th–75th percentile); *n* = number; CFS = Clinical Frailty Scale; ESI = emergency severity index, bpm = beats per minute, BP = blood pressure; mmHg = millimetres of mercury, NEWS = national early warning score; and ICU = intensive care unit.

**Table 2:** Odds ratios for the respective outcomes.

	Composite (30-Day-ICU-Admission or 30-Day-Mortality) (*n* = 51)			30-Day-Mortality (*n* = 27)			30-Day-ICU-Admission (*n* = 27)			Mechanical Ventilation (*n* = 7)			Admission to Medical Ward (*n* = 256)		
	OR	CI	P	OR	CI	P	OR	CI	P	OR	CI	P	OR	CI	P
Age, y	0.99	0.95–1.03	0.529	1.02	0.96–1.08	0.560	0.98	0.93–1.03	0.467	0.95	0.84–1.05	0.331	1.05	1.02–1.09	0.002
Female gender	1.21	0.64–2.27	0.554	1.28	0.55–2.98	0.568	1.17	0.51–2.64	0.711	0.95	0.18–4.55	0.947	0.62	0.38–1.01	0.055
Frailty (CFS > 4)	5.01	2.56–10.17	<0.001	6.92	2.75–19.54	<0.001	2.37	1.00–5.67	0.049	2.49	0.43–13.32	0.278	3.59	2.03–6.64	<0.001
COVID-19 positivity	3.47	1.48–7.89	0.003	3.54	1.14–10.16	0.021	3.41	1.24–8.63	0.012	7.10	1.31–35.4	0.015	1.32	0.65–2.79	0.457

The table comprises the odds ratios for the composite outcome (30-day-mortality or 30-day-ICU-admission), odds ratios for 30-day-mortality, for 30-day-ICU-admission, for mechanical ventilation, and for admission to a medical ward. The models were calculated for the CFS-category “frail” (CFS > 4) and for COVID-19-positivity with age and female gender as covariables. Non-frail-patients (CFS ≤ 4) and COVID-19-negative-patients were reference categories. OR = odds ratio; CI = 95% confidence interval; ICU = intensive care unit; and CFS = Clinical Frailty Scale.

## Data Availability

Data cannot be made open without written consent by the local ethics committee. Data sharing requests will be forwarded to the ethics committee. In case of acceptance of the request, the data can be shared in a fully anonymised form.

## Appendix A

**Table A1:** Patient characteristics: All potentially eligible patients compared to patients with missing CFS and patients analysed.

	All (*n* = 414)		Missing CFS (*n* = 42)		Analysed (*n* = 372)	
Age, median (IQR)	76.0	(71.0; 83.0)	71.0	(69.0; 78.8)	77.0	(71.0; 83.0)
Female gender, n (%)	168	(40.6)	14	(33.3)	154	(41.4)
30-day-mortality, n (%)	37	(8.9)	10	(23.8)	27	(7.3)
ESI level, n (%)						
1	18	(4.4)	7	(16.7)	11	(3.0)
2	210	(50.7)	15	(35.7)	195	(52.4)
3	164	(39.6)	7	(16.7)	157	(42.2)
4	6	(1.5)	2	(4.8)	4	(1.1)
5	0	(0.0)	0	(0.0)	0	(0.0)
NA	16	(3.9)	11	(26.2)	5	(1.3)

**Table A2:** Groups of final diagnoses of all 328 patients with suspected COVID-19 and subsequent negative SARS-CoV-2-PCR swab test.

	*n*	(%)
Post-COVID-19	1	(0.3)
Acute infection (non-SARS-CoV-2)	149	(45.4)
Pulmonary disease (non-infectious)	23	(7.0)
Cardiovascular disease	73	(22.3)
Neurologic disease	13	(4.0)
Psychiatric disease	8	(2.4)
Pain	14	(4.3)
Fall, trauma, rhabdomyolysis	10	(3.1)
Frailty syndrome	2	(0.6)
Electrolyte disorder	2	(0.6)
Other	33	(10.1)
